# Clinical Characteristics and Quality of Life in a Cohort of Polish Pediatric Patients with Hereditary Angioedema

**DOI:** 10.3390/children11020237

**Published:** 2024-02-13

**Authors:** Katarzyna Piotrowicz-Wójcik, Malgorzata Bulanda, Ewa Czarnobilska, Grzegorz Porebski

**Affiliations:** Department of Clinical and Environmental Allergology, Jagiellonian University Medical College, Botaniczna 3, 31-503 Krakow, Poland; katarzyna.piotrowicz-wojcik@uj.edu.pl (K.P.-W.); gosia.lesniak@uj.edu.pl (M.B.); ewa.czarnobilska@uj.edu.pl (E.C.)

**Keywords:** hereditary angioedema, HAE, children, C1 inhibitor deficiency, quality of life, pediatric population

## Abstract

Hereditary angioedema (HAE) is a rare genetic disease. It is characterized by recurrent attacks of angioedema. Evidence to what extent it affects patient functioning is limited in the pediatric population. We aimed to determine the clinical characteristics and management of Polish children with HAE and to measure the health-related quality of life (HRQoL) of these patients. This cross-sectional study was conducted among 21 pediatric patients and their caregivers, as well as 21 respective controls randomly selected from the general population. During routine follow-up visits, standardized pediatric quality of life questionnaires (PedsQL^TM^ 4.0) were administered to all caregivers and adolescents (≥13 years). Caregivers also completed a structured medical interview regarding the clinical characteristics and treatment of children with HAE during the previous six months. During this period, 57% of patients had low (group I), 24% moderate (group II), and 19% high (group III) HAE activity, corresponding to ≥10 attacks per 6 months. None of the patients received long-term prophylaxis. The children in group III had a lower HRQoL than other groups and controls on all dimensions of the PedsQL^TM^ 4.0. The lowest scores in all groups were observed in the emotional functioning domain. Our data demonstrate that the burden of HAE on the quality of life of pediatric patients and their families encompasses a wide range of daily functioning.

## 1. Introduction

Hereditary angioedema (HAE), caused by C1 inhibitor deficiency, is a rare genetic disorder with autosomal dominant inheritance, although de novo mutations occur in approximately 25% of cases. More than 50% of patients may become symptomatic before the age of 10 years [1]. Rarely, individuals with causative mutations remain asymptomatic, but most patients experience recurrent attacks with a variety of disabling symptoms and acute pain. Attacks usually affect the extremities, abdomen, and face. Attacks affecting the larynx are life-threatening. Hormonal changes, infections, or stressful situations can trigger attacks; however, numerous attacks happen due to an unidentified factor [2,3].

HAE has a significant impact on health-related quality of life (HRQoL) in patients of all ages [4,5,6]. According to studies conducted on adult patients, HAE interferes with everyday activities, limits work or school productivity, and extends to patients’ leisure time. The burden of HAE affects the time free from attacks, as patients report depression and worry about the future course of the disease, uncertainty about the timing of attacks, and inheritance risk in children [7]. Instruments specifically designed to assess HRQoL in HAE that are validated include the “Hereditary Angioedema Quality of Life Questionnaire” [8], the “HAE Patient-Reported Outcomes Questionnaire” [9], and the “United States HAE Association QoL Questionnaire” [10]. The “Angioedema Quality of Life Questionnaire” has also been shown to be an adequate and effective set of questions for describing HRQoL in a various population of adult patients with HAE [7]. In contrast to the adult population, there is a paucity of data on the HRQoL of children with HAE and their parents’ perceptions of the problem. In this study, we seek to improve this knowledge.

The aims of this study were (i) to determine the clinical characteristics and management of acute HAE attacks in Polish children with HAE; (ii) to evaluate quality of life in patients with hereditary angioedema using the Pediatric Quality of Life Questionnaire (PedsQL^TM^ 4.0) and to correlate it with the clinical course of the illness; (iii) to compare QoL reports between adolescent patients and their parents as reported in the PedsQL^TM^ 4.0 questionnaire—self-report for adolescents (13–18 years) and parent report; and (iv) to identify the most important problems in the home and hospital care of children with HAE as reported by their parents.

## 2. Materials and Methods

Children aged 5 to 18 years with a confirmed diagnosis of hereditary angioedema based on the recent guidelines [2,11,12] qualified for this study. During routine follow-up visits at the center, 33 patients, who are followed in our center, and their parents were invited to participate in this study. Of these, 21 confirmed their willingness to participate in this study and, after giving informed consent, were included in this study. They were administered a structured medical interview about HAE and a pediatric quality of life (QoL) questionnaire. This questionnaire was also addressed to the parents of 21 children randomly selected from the general population and matched to the study group in terms of sex and age. They formed the control group of this study. This study was conducted between April 2021 and October 2022 and was approved by the local Bioethics Committee (no: 1072.6120.37.2021).

A structured medical interview about HAE was addressed to the parents and administered during the visit. If this was not possible during the visit, it was conducted by telephone, or the parents responded by mail. The interview included basic anthropometric data, clinical history, signs and symptoms, and the treatment of HAE during the previous 6 months (disease-specific medications available to children during the study: icatibant for on-demand treatment, recombinant human C1 inhibitor (rC1 INH) for on-demand treatment, plasma-derived C1 inhibitor (pdC1 INH) for on-demand treatment and short-term prophylaxis, and lanadelumab for long-term prophylaxis). Based on this interview, patients were arbitrarily assigned to 3 groups according to HAE activity: group I (asymptomatic), group II (1–9 attacks/6 months), and group III (≥10 attacks/6 months).

The PedsQL^TM^ 4.0 was completed by the child’s caregiver and, if the child was at least 13 years old, by the child. The teenage patients and their parents completed the questionnaire in the waiting room of the center after the visit and were instructed to complete it independently. The PedsQL™ is a 23-item questionnaire that assesses physical, emotional, social, and school functioning across four domains. The physical functioning domain comprises eight items, while the others consist of five items. The parent proxy report used in the study includes young children, children, and adolescents (5–7, 8–12, and 13–18 years old, respectively) and assesses parents’ perceptions of their child’s QoL. The participants’ answers were measured on a 5-point Likert scale which ranged from 0 (there is never a problem) to 4 (this is always a problem). The items were then inverted and transformed linearly to a scale ranging from 0–100 (0 = 100, 1 = 75, 2 = 50, 3 = 25, 4 = 0). The total score was gained by summing points from all dimensions. The less points on the questionnaire, the better QoL of the subjects. We used the parent and child reports of the PedsQL^TM^ 4.0 Generic Core Scales for adolescents (13–18 years) and parent reports for children (8–12 years) and toddlers (5–7 years) [13].

We performed statistical analyses using the Julia programming language, version 1.9.4, and the following packages: HypothesisTests.jl, Statistics.jl, DataFrames.jl, and Pluto.jl. For statistical tests, the significance level was set at 0.05. The one-sample Anderson–Darling test was used to reject the null hypothesis that parental sex comes from the binominal distribution. Pearson’s correlation test was used to test whether there was a correlation between the groups analyzed and whether it was statistically significant. A one-way ANOVA test was used to test whether the means were equal between the groups for all the examined areas. An unpaired *t*-test for equal and unequal variances was performed to check significant differences between two groups for all examined areas. A graphical abstract was created using www.freepik.com (accessed on 22 January 2024).

## 3. Results

### 3.1. Study Group General Characteristics

Data were obtained from 21 children (12 girls and 9 boys) with a mean age of 10.6 years (range: 5–16, median: 11, SD: 4.1) (Table 1). There was a predominance of female caregivers who completed the questionnaire (17/21, 81%, *p* < 0.05). The control group consisted of 21 children who did not differ in sex and age from the study group (mean age of 10.71 years, range: 5–16, median: 11, SD: 3.7). The sex ratio of the parents was the same as in the study group. The mean age of becoming symptomatic was 4.9 years (range: 1–14, median: 4 years, SD: 3.4) (Table 2). Eleven children underwent diagnostic testing and were diagnosed with HAE in early childhood due to a family history of HAE. In the remaining children, the HAE diagnosis was delayed by 2.6 years (range: 0–8, median: 2 years, SD: 2.2). HAE type 1 was diagnosed in 18 patients (86%) and HAE type 2 in three patients (14%). A positive family history of the disease was present in 18 patients (86%). A HAE-related death among the patients’ family was reported by five patients (24%). 

Nine of the twenty-one patients had HAE symptoms within 6 months preceding the survey, but 18 patients from the entire study group were symptomatic for a longer period of time. Most attacks of HAE affected one body area at a time, but three patients reported more than one localization at a single time point. The most common HAE symptoms within the 6 months prior to the survey were peripheral swelling in seven patients (59 attacks in total), abdominal attacks in six patients (38 attacks), facial swelling in four patients (14 attacks), urogenital attacks in three patients (six attacks), and laryngeal attacks in two patients (five attacks).

Patients with an earlier age at first HAE attack tended to have more attacks during last 6-month period, but this association was not significant. The mean duration of an HAE attack was 3.3 days (range: 2–6, SD: 1.4, median: 3 days). The patients’ caregivers reported many different trigger factors: infections (six patients), emotional stress (five patients), trauma (injury) (five patients), exertion (five patients), menstruation (two patients), strong emotions (five patients), medical procedures (two patients), pressure (three patients), and cold (one patient). Six patients reported prodromal symptoms—erythema marginatum (two patients), pain (two patients), itch (one patient), anxiety (two patients), and weakness with a subjective sensation of impending fainting (one patient). The first episode of HAE was localized to the abdomen in 50% of symptomatic patients, to the extremities in 33%, and to the face in 17%.

All patients had a home supply of emergency rescue medication: plasma-derived C1-INH (20 patients), icatibant (nine patients), and recombinant human C1-INH (rhC1-INH) (one patient). Most parents bring along an emergency medication for their children while traveling (19 persons). During a severe HAE attack, only nine parents were able to administer a drug themselves (icatibant—five; pdC1-INH—four). Thirteen patients were taken to hospitals or outpatient clinics during an HAE attack where they were given rescue medication (pdC1-INH—13 patients; icatibant—one).

A total of six out of nine patients who had been symptomatic for the previous 6 months received on-demand treatment. PdC1-INH only was used in three patients, pdC1-INH and icatibant in two patients, and tranexamic acid in one patient. All laryngeal attacks were treated (two patients, five attacks in total), most abdominal (four of six patients, 22 attacks in total) and facial (three of four patients, 13 attacks in total). Peripheral attacks were treated in two of seven patients (a total of six attacks). No urogenital attacks were treated (three patients, total of six attacks). Tranexamic acid had been used by three patients and danazol by one patient in the past, but the efficacy of these drugs was low. In the 6 months prior to the survey, none of the patients had received long-term prophylaxis (LTP), and short-term prophylaxis (STP) was used by two patients.

### 3.2. Everyday Problems from Parents’ Perspective

HAE was a reason for absence from kindergarten or school in eight symptomatic children for a total of approximately 109 days, representing approximately 1100 h of lost activity. Four patients had to reduce their sports activities because they provoked attacks. Six parents reported travel problems due to frequent or unpredictable HAE attacks in their children. During the interview, the parents were asked open-ended questions about additional problems they noticed in dealing with their children with HAE. The parents had many comments and problems that they often encounter, which are presented below. We divide them into two categories: dependent (A) and independent (B) of health care, as shown in Table 3.

### 3.3. Quality of life—PedsQL^TM^ 4.0

#### 3.3.1. General Quality of Life among HAE Children (Parents’ Proxy Report)

The most affected area of life in children with HAE was emotional functioning (mean: 71.43), which includes feelings of anxiety, sadness, anger, difficulty sleeping, and uncertainty about the future; data are presented in Table 4. The mean quality of life scores did not differ between the study group and controls (Table 4).

#### 3.3.2. Correlation between Parent-Reported QoL and Disease Activity

To investigate the relationship between QoL and disease activity, we divided the patients into three groups as described above. Group I, group II, and group III corresponded to low, moderate, and high HAE activity. There were 12 patients in group I, five in group II, and four in group III. Higher disease activity was significantly correlated with a lower QoL regarding physical, emotional, social, school-related, psychosocial, and total QoL based on the parents’ proxy reports. The most affected area of QoL in each group of patients was emotional functioning (Table 5). Group III included only girls, with a mean age of 14 years (range: 9–16 years). The mean quality of life scores did not differ between study groups II and III and the control group, but were significantly lower in all domains in group III compared with the control group (*p* = 0.01 for domain “school-related”, *p* < 0.01 for all other domains).

#### 3.3.3. Comparison of QoL Reported by HAE Adolescents and Their Parents

The group of adolescent patients included eight individuals (five female, three male). The mean age of the group was 14.9 years (range: 13–16). There were four females and four males in the parent group, with a mean age of 44.4 years (range: 38–54). No significant differences were observed between the patient and parent versions of the PedsQL^TM^ 4.0 in any of the domains examined (Table 6). When comparing the reports of parents of symptomatic and asymptomatic children, significant differences were found in the physical domain, but not in other domains (Table 7).

## 4. Discussion

This study included 21 patients aged 5 to 16 years with a diagnosis of HAE, predominantly HAE type 1. Most of our patients have a family history of HAE (86%) and 11 children were diagnosed in early childhood. Following international guidelines, in our center, we encourage all patients with HAE to test their children, even if they are asymptomatic. Until a full diagnosis is made, children of patients with hereditary angioedema should be considered to have HAE [12]. The mean age at the onset of symptoms in our study was 4.9 years, which is much lower than the previous description of the Polish adult population (13 years) [14]. The age of the first HAE attack is negatively correlated with the number of attacks in the last 6 months. In fact, when HAE symptoms appear in a younger child, it may be associated with a more severe clinical course [12]. In our study, the first HAE attack was localized in the abdomen in 50%, in the extremities in 33%, and in the facial area in 17% of symptomatic patients. In a Danish study, the first attack involved the limbs in 8/14 children and the abdomen in 6/14 children. No patient had a first attack in the upper airways [15].

Early diagnosis, education, and provision of rescue medication to the patient are critical, because although laryngeal attacks in children are very rare, they can be life-threatening. The delay in HAE diagnosis was 2.6 years in children without a family history of HAE. Our results are comparable to Brazilian pediatric data from 2021, where 84% of patients had a family history and the mean delay to diagnosis was 3.9 years [16]. In contrast, the mean delay to diagnosis in adult patients in Poland was 15.2 years [14], which is in line with observations from other adult cohorts: Switzerland (mean: 14 years in patients with a negative family history) [17], the United Kingdom (HAE type 1—10 years; HAE type 2—18 years) [17], Greece (16.5 years) [18], Sweden (10 years) [19], Korea (7.8 years) [20], and the United States of America (8.4 years) [4]. The increased awareness of HAE in recent years has led to a shorter time to diagnosis in younger patients, as observed in the previous study [14].

In our study, 9 out of 21 patients had symptoms within the previous 6 months; 18 patients were symptomatic for a longer period of time. Patients reported many typical triggers of HAE attacks: infections, stress, physical trauma, exertion, menstruation, strong emotions, medical procedures, pressure, and cold. Unexpectedly, no child mentioned food as a trigger, which has been reported in some adult studies [14,21]. This may be due to the relatively small number of patients surveyed. A few of our patients reported prodromal symptoms, including erythema marginatum in only two cases. According to the literature, erythema marginatum occurs in 42% to 58% of children with HAE but is often confused with urticaria. Martinez-Saguer and Farkas reported the onset of severe and recurrent erythema marginatum in two newborns. In these cases, prodromal symptoms made it easier to establish the diagnosis of HAE, so that future angioedema attacks could be treated quickly and correctly [22]. The presence of prodromal symptoms may help to recognize an attack and initiate early treatment.

All our patients had a home supply of rescue medications for use in emergency situations: pdC1-INH, icatibant, or rhC1-INH. However, during a severe HAE attack, only a minority of parents were able to administer medication to their children. In Denmark, all symptomatic children were treated with tranexamic acid and/or pdC1-INH. Androgens were not used as in our group of patients within the last 6 months before the survey. Home therapy with pdC1-INH was given in 9 of 22 cases: six children were trained in self-administration and three children were treated by their parents [15]. The inability to obtain medication at home during an attack implies the need to seek medical assistance and delays treatment. These data demonstrate the continuing need for injection training for parents and adolescent patients. In the study by Prenzel et al. from 2016 to 2021 in Germany, there was a considerable increase in prescriptions of icatibant for on-demand treatment. The authors explained this trend based on the permission to prescribe icatibant for children ≥ 2 years in 2017 and the ease of use of the subcutaneous pre-filled syringe [23].

In our study, patients did not receive long-term prophylaxis (LTP). The reason may be the limited availability of disease-specific LTP in Poland, due to restrictive criteria authorizing the prescription of such treatment. International guidelines recommend pdC1-INH, lanadelumab, or berotralstat as first-choice LTP [2]. In our country, only lanadelumab is available to patients over 12 years of age after meeting strict criteria, such as a documented frequent occurrence of severe angioedema attacks—at least 12 life-threatening attacks (abdomen, larynx, or throat) with use of rescue medication within the last 6 months. The second-line preventive drug, danazol, is not recommended for use in children until they have finished growing and has numerous side effects [12]. In a German study, the number of patients on LTP treatment increased from 7.0% (2016) to 27.9% (2021) [23]. The LTP treatment rate in a recent French study was significantly higher (59.2%) [24]. Similarly, in a 2014 UK study of 111 children, about 30% of patients were on LTP [25]; in a later study, 45% of patients were on LTP [26]. However, a large number of these patients received androgens and progestins [25,26]. The proportion of children using LTP in the global HAE registry was 8.6%, two-thirds of whom were treated with tranexamic acid [27]. Therefore, although tranexamic acid and danazol are sometimes used in LTP, usually depending on the local situation and the availability of modern drugs, current guidelines indicate that HAE-specific medications are considered the proper LTP for HAE. [2]. Short-term prophylaxis with pdC1-INH was used in two patients during the observation period. Sometimes, drugs such as androgens and tranexamic acid are used off-label for STP, possibly due to limited access to certain drugs [16].

HAE attacks can cause children to miss day care or school, reduces physical activity, and sometimes causes problems with eating or traveling. Another problem is the lack of understanding among teachers and other students, despite detailed information about the disease. School or kindergarten staff are often afraid of what to do in the event of an HAE attack. All of these factors can significantly affect the quality of life of a group of pediatric HAE patients.

Previous studies assessing QoL in pediatric HAE patients were conducted by Nygren et al. [28] and Engel-Yeger et al. [29]. The study by Nygren et al. included 36 Swedish children aged 1–17 years with HAE, with a parental assessment of the child’s QoL during the previous week using a single-item seven-point visual analog scale for facial expressions [28]. The study by Engel-Yeger et al. included 98 children (34 HAE patients, 64 healthy controls) aged 3–18 years from Hungary and Israel. All subjects completed a disease activity and location questionnaire, a demographic questionnaire, and the PedsQL^TM^ 4.0 (child self-report and maternal proxy report) to describe their health-related quality of life [29].

The most affected domain of life in our group of children with HAE was emotional functioning (mean: 71.43), which includes feelings of fear, sadness, anger, sleep problems, and uncertainty about the future, which correlates with the results of the Israeli and Hungarian study [29]. This finding may be related to the fact that HAE is an unpredictable disease, especially in patients who do not use long-term prophylaxis. Children affected by this disease, even those who are asymptomatic, are often worried about their future; they are afraid of the unpredictability of the disease and that a severe attack of swelling may occur unexpectedly, even in someone who has been completely asymptomatic, which is why the emotional domain is most affected in this group of patients. Other domains—physical, social, and school-related—are most affected during HAE attacks. Nygren et al. also used other survey tools and reported that the most affected HRQoL dimensions were pain, discomfort, anxiety, depression, shame, energy, fatigue, mood, and general health [28].

To investigate the relationship between QoL and disease activity, we divided our patients into three groups with low, moderate, and high HAE activity. We found that higher disease activity was significantly associated with a lower quality of life in several domains of functioning—physical, emotional, social, school-related, psychosocial, and total QoL—based on parent proxy reports. Further, we observed that the control group did not differ from children with HAE with low disease activity (group I), and when compared with the group with moderate disease activity (group II), there was a trend in favor of the control group in terms of quality of life, but the difference was not significant. In contrast, children with HAE with high disease activity (group III) showed a significant and statistically significant decrease in QoL when compared to controls. Considering that group I had no HAE attacks in the last 6 months and group III had 10 or more attacks, the above results seem reasonable and reflect the condition of the subjects. In the study by Engel-Yeger et al, the number of HAE attacks was also negatively correlated with the total HRQoL score, school-related HRQoL, and psychosocial HRQoL [29]. In group III, consisting of adolescent girls, the severity of the disease can be attributed to physiological hormonal changes. Ovulation and menstruation usually exacerbate the course of the disease and make it more difficult to differentiate abdominal pain [30,31]. 

In our study, there were no significant differences between the child and parent versions of the PedsQL ^TM^ 4.0 in all domains examined. Similarly, in the study by Ocak et al. [32], a significant correlation was observed between self-reported and parent-reported HRQoL in total and in all subscales in patients with HAE. The authors recruited 60 children with histaminergic angioedema (HA), 59 children with HAE, and 72 healthy controls. The total HRQoL score and other subscale scores of the healthy controls were higher than in the patients with HAE and the patients with HA [32]. The difference in the physical domain found when comparing parents’ reports of quality of life in symptomatic and asymptomatic children with HAE may be surprising at first (Table 7). This may be because two respondents in the “symptomatic” group scored very low in the “physical” domain (28 and 31 points), which, given the small sample size, lowered the overall score. This highlights the fact, observed in daily contact with HAE patients, that the perception of the disease can vary greatly between individuals, from people who lead a fully active professional and social life to those who perceive their disease at a similar level of severity as being a serious burden on daily functioning. In addition, the pressure or trauma sometimes associated with physical activity can induce HAE attacks. These respondents may have anticipated such a situation in their answers.

A limitation of our study is the arbitrary classification of HAE activity according to attack frequency. The HAE activity scale used in adults (Angioedema Activity Score) [33] was not originally tested on children, but the number of HAE attacks in an assessed period of time is a clinical parameter used, for example, in clinical trials as an important criterion for assessing disease severity and potential drug response. The relatively small study sample is related to the obvious fact that HAE is a rare disease, and we studied an age subgroup from a single center. In addition, up to on-third of caregivers of children with HAE did not want to participate in the study, which may be explained by their reluctance to complete questionnaires about their children and not directly related to treatment. Five years of age being the lower age limit of the study subjects was due to the consent conditions set by the bioethics committees. The HRQoL tool used (PedsQL^TM^ 4.0) is not specific to HAE or angioedema in general. However, existing popular questionnaires such as AE-QoL [34] have not been validated with children and adolescents.

## 5. Conclusions

Given the limited data from the pediatric population, our results provide novel evidence on the burden of HAE on the quality of life of children and their families. The perception of the disease as a factor affecting daily life may be influenced by variables such as education level, the availability of medications, and access to healthcare professionals specializing in HAE. Our data provide further insight into an unmet medical need of HAE patients, namely the low number of HAE patients who self-medicate. For many years, only emergency treatment was available to stop a life-threatening angioedema attack. Currently, the new treatment paradigm for HAE emphasizes LPT and the achievement of complete symptom control. In addition, new drugs for LTP are being researched that may cover pediatric age groups [2] and help to improve the quality of life of children with HAE.

## Figures and Tables

**Table 1:** Demographics of children with HAE and their parents.

Characteristic	The Study Group (Children)	Parents
Age, years Mean Min, max	10.6 5, 16	40.4 28, 54
Sex, n (%) Female Male	12 (57%) 9 (43%)	17 (81%) 4 (19%)
Place of residence Village Small city City ^1^	8 (38%) 4 (19%) 9 (43%)	8 (38%) 4 (19%) 9 (43%)

^1^ More than 200,000 inhabitants.

**Table 2:** Clinical characteristics of children with HAE.

Characteristic	The HAE Group (Children)
Type of HAE, n (%) Type 1 Type 2	18 (86%) 3 (14%)
Positive family history, n (%) yes no	18 (86%) 3 (14%)
Age at disease onset, years Mean Min, Max	4.9 1, 14
Time from first symptoms to diagnosis ^1^, years Mean Min, Max	2.6 0, 8

^1^ Refers to children not tested due to family history of HAE in early childhood.

**Table 3:** HAE-related problems reported by parents in open-ended questions.

Health Care-Dependent Area	Independent of Health Care QoL—Mean
Insufficient knowledge of HAE in primary care physicians	Fear of laryngeal attack with any upper respiratory infection
Strong need for training in the administration of rescue medication (especially pdC1-INH) for parents and medical staff	Inability to go on independent trips such as summer camps due to inability to administer pdC1-INH quickly
The need for prompt admission of patients with HAE attacks and administering the drug in primary care, and the need to administer the drug in the emergency room without admission to the ward	Lack of understanding among teachers despite detailed information about the disease and fear of school/kindergarten staff about what to do in the event of an HAE attack
The need for LTP in children	Parental fear of increased frequency of HAE attacks
Insufficient psychological support for young people	Need to socialize with other patients living normal lives
Failure to inform patients about the possibility of obtaining a disability certificate	Sudden HAE attacks in a child requiring parents to leave work suddenly to provide rapid rescue treatment

**Table 4:** Overall quality of life in patient group and controls.

Area	QoL—Mean (SD ^1^) HAE Group	QoL—Mean (SD ^1^) Controls	*p*
Physical	83.78 (21.82)	91.96 (9.25)	0.58
Emotional	71.43 (27.21)	82.62 (14.72)	0.25
Social	86.67 (20.39)	96.19 (7.23)	0.08
School-related	84.25 (22.09)	88.81 (12.74)	0.47
Psychosocial	79.84 (22.4)	89.21 (8.88)	0.41
Total QoL	81.21 (21.41)	90.18 (8.24)	0.35

^1^ standard deviation.

**Table 5:** Quality of life in patients with low (group I), moderate (group II), and high (group III) disease activity.

	Group I	Group II	Group III	
Area	Mean (SD)	Mean (SD)	Mean (SD)	*p*
Physical	96.35 (6.08)	80.62 (15.37)	50 (23.52)	<0.001
Emotional	82.08 (17.25)	73 (13.42)	37.5 (32.02)	0.004
Social	93.75 (11.1)	89 (13.41)	62.5 (33.04)	0.019
School-related	89.17 (13.79)	87 (13.03)	51.25 (28.69)	0.004
Psychosocial	88.33 (12.73)	83 (15.78)	50.42 (30.95)	0.006
Total QoL	91.12 (9.31)	82.17 (14.12)	50.27 (28.32)	0.001

**Table 6:** Comparison of quality of life reports from children with HAE and their parents.

	Children	Parents	
Area	Mean (SD)	Mean (SD)	*p*
Physical	74.22 (27.89)	75.39 (30.41)	0.937
Emotional	70.00 (33.06)	63.75 (37.3)	0.728
Social	81.87 (26.58)	81.87 (29.63)	0.858
School-related	78.75 (30.56)	76.25 (31.37)	0.874
Psychosocial	76.87 (29.55)	73.96 (32.37)	0.854
Total QoL	75.95 (28.35)	74.46 (30.89)	0.921

**Table 7:** Parents’ reports on quality of life in symptomatic and asymptomatic children with HAE.

QoL Reported from Parents	Symptomatic Children	Asymptomatic Children	
Area	Mean (SD)	Mean (SD)	*p*
Physical	51.56 (25.32)	99.22 (1.56)	0.0329
Emotional	45 (42.43)	82.5 (22.55)	0.1934
Social	66.25 (37.05)	97.5 (5.0)	0.1932
School-related	60 (38.08)	92.5 (11.9)	0.2018
Psychosocial	57.08 (38.91)	90.83 (13.09)	0.1987
Total QoL	55.16 (34.11)	93.75 (8.34)	0.1154

## Data Availability

The data that support this study are available from the corresponding author on request. The data are not publicly available due to privacy and ethical restrictions.
